# Snapshot 3D at the speed frontier: redefining light-field microscopy for neuroimaging

**DOI:** 10.1186/s43074-026-00265-z

**Published:** 2026-07-01

**Authors:** Ruixuan Zhao, Jongchan Park, Liang Gao

**Affiliations:** 1 Department of Bioengineering, University of California, Los Angeles, CA USA; 2 California NanoSystems Institute, University of California, Los Angeles, CA 90095 USA

**Keywords:** Light-field microscopy, Neuroimaging, Calcium imaging, Voltage imaging, Computational microscopy

## Abstract

Neural activity unfolds across three-dimensional circuits on millisecond-to-microsecond timescales, yet most optical microscopes still acquire volumes sequentially, limiting their ability to capture fast, distributed dynamics. Light-field microscopy (LFM) addresses this unmet need by encoding spatial and angular information into a single camera exposure, enabling snapshot volumetric imaging with low latency and strong robustness to motion. Here we review emerging advances in light-field neuroimaging, from brain-wide calcium recordings in freely moving animals to recent progress that brings kilohertz-class volumetric voltage imaging within reach. We argue that LFM should be evaluated based on information throughput, latency, photon efficiency, and motion robustness at the speed frontier, but not as a direct resolution or contrast competitor to confocal, multiphoton, or light-sheet microscopy. We conclude by highlighting future directions that preserve the LFM’s snapshot advantage, including speed-preserving improvements in image quality, extreme temporal-bandwidth architectures that prioritize quantitative inference over visual appearance, and multimodal light-field sensing that adds spectral, lifetime, and polarization contrast.

## Highlights

- Light-field microscopy (LFM) enables snapshot capture of three-dimensional neural dynamics, shifting evaluation from “best resolution per voxel” to “best information per unit time.”
- In neuroimaging, LFM is established for fast calcium imaging and is emerging as a promising approach for volumetric voltage imaging, where the speed requirement is even more demanding.
- LFM’s snapshot acquisition has inherent structural trade-offs, including étendue division and tomographic sampling, which define both where LFM wins and where it should not compete.
- Next-generation LFM will be shaped by speed-preserving technologies that improve image quality, extend temporal bandwidth, and expand functional contrast.

## Introduction: why the “speed frontier” matters for brain imaging

Neural computation is fundamentally four-dimensional: activity propagates through three-dimensional (3D) circuits on millisecond-to-microsecond timescales while animals move, sense, and act. Optical neuroimaging is, therefore, being pulled in two directions at once: toward larger volumes (multi-region and brain-wide recordings) and toward faster signals (from population calcium transients to millisecond-to-microsecond voltage events). Yet most microscopes still acquire volumes sequentially, causing transient events to be sampled at different effective times across the field of view (FOV) and depth. This temporal misalignment complicates the interpretation of causality, synchrony, and closed-loop readouts.

Confocal and multiphoton microscopes provide optical sectioning but sample points serially. Light-sheet methods can acquire planes rapidly when sample geometry allows, but volumetric imaging still requires stepping through depth or sweeping a light sheet. As target volumes expand and relevant timescales shorten, sequential sampling can introduce temporal skew and motion artifacts, miss sparse transient events, and demands higher photodose to maintain signal-to-noise ratio (SNR). These costs are especially acute in awake, behaving animals, where motion correction and system latency can be as important as nominal spatial resolution.

Snapshot volumetric imaging changes the experimental calculus by making synchrony the default. In light-field microscopy (LFM), a microlens array (or equivalent angular encoder) maps spatial and angular information onto a 2D detector, and computationally reconstructs a 3D volume from each exposure [1–4]. This snapshot capability comes with structural trade-offs: the imaging objective’s available aperture, or optical étendue, is divided among angular samples, and information is acquired tomographically with nonuniform sampling in spatial-frequency space. Rather than viewing these constraints simply as limitations, we emphasize that they define the regimes where LFM is most compelling. At the speed frontier, the right evaluation metrics shift from “best resolution per voxel” to “best information per unit time”.

In this Perspective, we survey how LFM is being used, and re-engineered, for neuroimaging, with a focus on temporal throughput. We summarize emerging applications in calcium imaging and highlight volumetric voltage imaging as a particularly compelling use case for snapshot acquisition. We then position LFM alongside confocal, multiphoton, and light-sheet microscopy using a speed-resolution-contrast framework that clarifies complementary operating regimes. Finally, we outline technology directions that preserve LFM’s snapshot advantage while improving image quality, temporal bandwidth, and functional contrast.

## Emerging applications of light-field imaging in neuroimaging

### Calcium imaging

LFM gained traction in neuroscience by enabling brain-wide activity readouts in optically accessible organisms without the time cost of axial scanning. This snapshot advantage is especially compelling for capturing fast, distributed dynamics during behavior. Early demonstrations established snapshot whole-brain calcium imaging in immobilized larval zebrafish and C. elegans [3, 5]. More recent work has shifted the field from proof-of-principle demonstrations toward practical functional imaging in more challenging biological settings, including deeper tissue, larger FOVs, and freely moving animals, while also integrating imaging more tightly with behavioral readouts and perturbations.

**Fig. 1:**
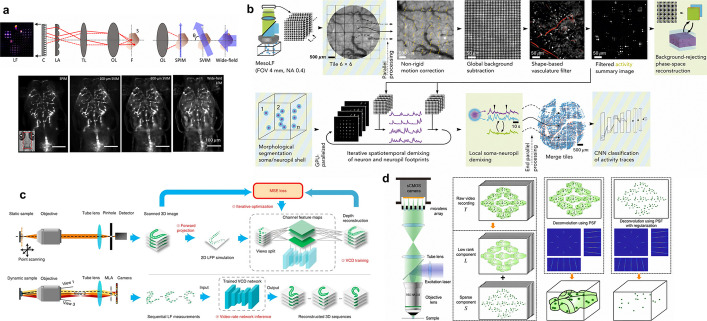
Light-field calcium imaging. **a** Selective volume illumination microscopy (SVIM) combines confined illumination with light-field detection to suppress out-of-volume background, improving image contrast while preserving synchronous snapshot volumetric imaging. The lower panels compare zebrafish brain-wide calcium imaging under single-plane illumination, slab illumination with varying thickness, and conventional wide-field flood illumination. LF, light-field; C, camera; LA, lenslet array; TL, tube lens; OL, objective lens; F, focus; S, sample; SPIM, single-plane illumination microscopy. **b** Mesoscale volumetric light-field microscopy (MesoLF) extends calcium LFM to scattering mammalian cortex, using a large-FOV optical design and computational pipeline for motion correction, background subtraction, demixing, tile merging, and activity-trace classification. **c** View-channel-depth network light-field microscopy (VCD-LFM) uses supervised deep learning to accelerate volumetric reconstruction, reducing reconstruction latency and enabling real-time recovery of dynamic 3D biological activity from multiplexed light-field measurements. **d **Sparse decomposition light-field microscopy improves source separation and denoising by decomposing light-field measurements into low-rank and sparse components, supporting high-speed extraction of neuronal activity from volumetric recordings. Figures adapted from Refs [6, 8, 11, 13]

On the optical side, a key advance has been the integration of selective volume illumination microscopy (SVIM) [6] with LFM (Fig. 1a). By suppressing out-of-volume background while preserving snapshot acquisition, SVIM substantially improves effective contrast for functional imaging in vivo. Single-objective SVIM further extends this strategy to conventional microscope platforms, making high-contrast light-field calcium imaging more accessible to neuroscience laboratories while maintaining volumetric imaging speed [7]. In parallel, mesoscale volumetric light-field imaging (MesoLF) [8] has extended LFM to scattering mammalian cortex, enabling near-simultaneous calcium imaging of thousands of neurons across multi-millimeter cortical fields in real time (Fig. 1b). This work is especially important because it expands LFM from optically transparent model systems toward large-FOV circuit imaging in scattering mammalian brain tissue.

On the computational side, learning-accelerated reconstruction has addressed one of the central bottlenecks of LFM: converting highly multiplexed 2D measurements into interpretable 3D activity maps fast enough for experimental use. By reducing reconstruction latency from minutes to near real time, these methods enable interactive experiments, online quality assessment, and closed-loop analysis. Recent physics-driven and self-supervised frameworks, such as SeReNet [9] and HyperNet [10], further improve generalization and processing speed, providing millisecond-level reconstruction and increased robustness to noise, aberrations, and sample motion. On the other hand, learning-based approaches continue to improve reconstruction quality for downstream functional inference. For example, Deep-learning-based LFM reconstruction [11] (Fig. 1c) and continuous-validation strategies [12] that reduce reconstruction artifacts and drift have both helped turn high-volume-rate raw measurements into stable functional readouts. Complementary model-based approaches, such as sparse decomposition, further improve neuron separability and denoising for real-time neural activity analysis [13] (Fig. 1d).

On the application side, LFM is increasingly being used not simply to image larger neuronal populations, but to address circuit-level questions that specifically require volumetric simultaneity. These include distributed brain-state dynamics, whole-brain coordination, and rapid population transitions that would be temporally blurred or misregistered by sequential scanning. For example, fast near-whole-brain LFM in behaving adult *Drosophila* revealed global activity changes associated with walking, grooming, sensory responses, and spontaneous activity patterns [14], while subsequent whole-brain calcium imaging linked spontaneous and forced locomotor behaviors to brain-wide state changes [15]. More recently, all-optical LFM in freely swimming larval zebrafish combined brain-wide calcium imaging with real-time 3D tracking, rapid reconstruction/registration, and targeted optogenetic stimulation, enabling causal interrogation of distributed neural circuit dynamics during naturalistic behavior [16].

Taken together, these optical, computational, and application-driven advances are steadily raising the practical ceiling of calcium LFM while retaining the key advantage that motivated LFM in the first place: snapshot volumetric sampling at behaviorally relevant speeds.

### Voltage imaging

Unlike calcium imaging, which infers neural electrical activity indirectly through intracellular calcium dynamics, voltage imaging with genetically encoded voltage indicators (GEVIs) or voltage-sensitive dyes (VSDs) provides a more direct readout of membrane potential. However, the relevant signals, including action potentials, dendritic spikes, and fast synaptic transients, occur on timescales that push conventional volumetric microscopes to their limits [17–19]. This is where snapshot LFM is especially well matched: by trading some image quality for simultaneous 3D sampling, it can access kilohertz-class volumetric rates that are difficult to achieve with sequential scanning. Recent in vivo demonstrations of high-speed voltage imaging underscore both the promise of optical voltage readout and severe temporal-bandwidth constraints that motivate snapshot 3D imaging [20, 21].

**Fig. 2:**
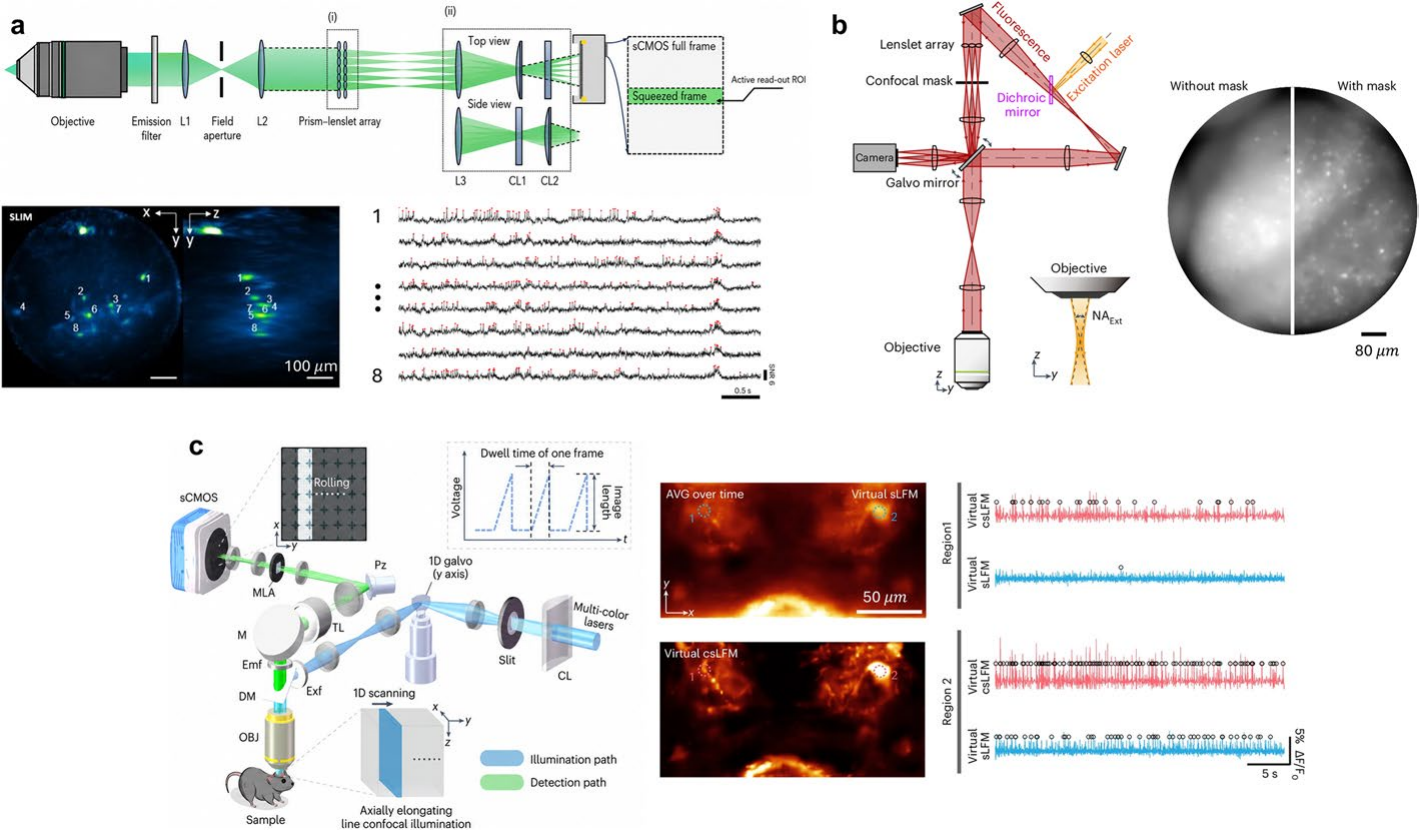
Light-field voltage imaging. **a** Squeezed light-field microscopy (SLIM) uses anisotropic light-field encoding to compress volumetric information into a reduced camera readout region, enabling kilohertz-class three-dimensional imaging of neural dynamics; Representative reconstructions and fluorescence traces demonstrate volumetric spike readout from the in vivo mouse hippocampus. **b** Confocal light-field microscopy combines light-field detection with confocal background rejection to improve contrast for volumetric voltage imaging in the mouse brain; The example compares cortical imaging with and without the confocal mask, showing improved source visibility after background suppression. **c **Confocal scanning light-field microscopy (csLFM) combines line-confocal illumination with rolling-shutter readout of a scientific CMOS camera to enable optically sectioned light-field detection; Representative images and traces illustrate improved image contrast in the time-averaged image and functional readout compared with conventional virtual scanning light-field microscopy (sLFM). MLA, microlens array; Pz, piezoelectric actuator; TL, tube lens; M, mirror; Emf, emission filter; DM, dichroic mirror; OBJ, objective lens; Exf, excitation filter; CL, cylindrical lens; AVG, average. Figures adapted from Refs [23, 27, 28]

Multiple recent studies have demonstrated that LFM can support functional voltage readout across 3D volumes. In acute mouse brain slices, LFM has been shown to localize GEVI signals in 3D with subcellular resolution, including dendritic voltage transients, without sacrificing SNR relative to widefield imaging [22]. Squeezed light-field microscopy (SLIM) [23] pushes this concept into behaving mammals, demonstrating volumetric voltage spike and subthreshold oscillation readout at kilohertz-class rates in vivo (Fig. 2a). Meanwhile, continued advances in indicator engineering, including positively tuned voltage reporters with improved dynamic range and robustness [24, 25], are poised to further expand the practical operating space of volumetric voltage imaging.

Two complementary trends are helping bridge the remaining gap between “possible” and “routine” voltage LFM in brain tissue, where scattering and aberrations must be addressed. First, hardware strategies such as confocal detection [26–28] (Fig. 2b and c) and selective volume illumination [23] improve background suppression by confining either signal collection or excitation to the volume of interest [21–23]. Second, computational approaches, including digital adaptive optics [29, 30] (DAO) and volumetric scattering microscopy [31] (VSM), can correct aberrations arising from tissue refractive-index heterogeneity, thereby improving image sharpness and signal localization at depth.

Even with these advances, the imaging depth of LFM in brain tissue remains fundamentally constrained by light diffusion. For one-photon excitation, the practical depth limit is typically on the order of one transport mean free path, approximately 1 mm. To reach deeper regions, a promising direction is to integrate LFM with wide-field two-photon excitation strategies, such as holographic two-photon microscopy [32] or temporal-focusing two-photon microscopy [33]. By using longer-wavelength excitation, these approaches could extend penetration depth while preserving the parallel volumetric detection advantage of LFM.

Another frontier in voltage LFM is efficient data capture. Unlike calcium imaging, voltage imaging typically requires kilohertz frame rates, which can generate enormous data volumes even over moderate recording durations. This creates challenges not only for data transfer and storage, but also for downstream reconstruction and inference. These data-bandwidth demands are one reason voltage LFM has not yet become as widely adopted as calcium LFM.

Compressive LFM offers a promising route to address this bottleneck by reducing data load before camera readout. SLIM [23], for example, compresses information in the spatial domain through anisotropic image squeezing, whereas EventLFM [34] replaces frame-based acquisition with asynchronous event readout. Because compression occurs at the acquisition stage, these approaches reduce the data burden at its source. This strategy is particularly well matched to action-potential imaging, where neuronal firing events are intrinsically sparse in both space and time.

A more aggressive direction is to move beyond conventional microlens-array architectures and use end-to-end optical-computational co-design to optimize the light-field encoder itself, for example through phase plates or random microlens arrays [35–37]. Such approaches could improve capture efficiency by tailoring the optical measurement to the expected sample structure and inference task. However, they also rely more strongly on structural or temporal sparsity priors, making them best suited to sparsely labeled samples.

It is worth noting that, beyond neuronal calcium and voltage readouts, LFM has also been applied to fast vascular dynamics, including volumetric measurements of cerebral blood flow [23, 38]. These studies broaden the relevance of LFM for neuroimaging by linking fast neural activity to vascular and hemodynamic physiology.

### Positioning LFM in the broader landscape of 3D optical microscopy

LFM sits in a crowded palette of 3D bio-optical imaging tools. Confocal and multiphoton systems remain the gold standard when the primary goal is spatial resolution and optical sectioning in scattering tissue. Two-photon innovations such as light-beads microscopy [39] and mesoscopes [40] have expanded sequential volumetric imaging to higher rates and larger FOVs. However, they still sample volumetric content over time and therefore face trade-offs when dynamics are fast and spatially distributed. On the other hand, light-sheet microscopy often offers the best overall compromise—high contrast with reduced phototoxicity and fast volumetric rates when sample geometry allows. SCAPE-style methods exemplify how plane acquisition can reach volumetric rates that point-scanning approaches cannot, while maintaining strong optical sectioning for many sample configurations [41, 42]. Because these modalities are structurally advantaged along the image quality axis, it is not strategically sound to position LFM as another route to confocal- or light-sheet-like quality. That framing invites an unfair comparison on the dimension where LFM is not designed to win.

The value proposition of LFM is fundamentally different: snapshot 3D favors synchrony, motion robustness, and low latency. Rather than pursuing best resolution or contrast through sequential point or plane scanning, LFM distributes measurement resources across a volume in a single exposure, yielding high information throughput when dynamics are fast or behavior-linked. This throughput-first trade-off is well matched to neural processes where timing and causality matter (e.g., transient population events and voltage spiking signals) and to experiments that demand real-time readout or closed-loop control.

This positioning is also consistent with LFM’s structural costs. Snapshot speed is achieved by dividing available imaging objective’s aperture, or optical étendue, across angular samples, reducing effective spatial resolution and contrast relative to spatial scanning. Moreover, LFM is inherently tomographic: information is acquired with nonuniform sampling in spatial-frequency space, typically oversampling low frequencies while undersampling high frequencies. These constraints are not merely limitations; they define the regimes in which LFM is most appropriately deployed.

**Fig. 3:**
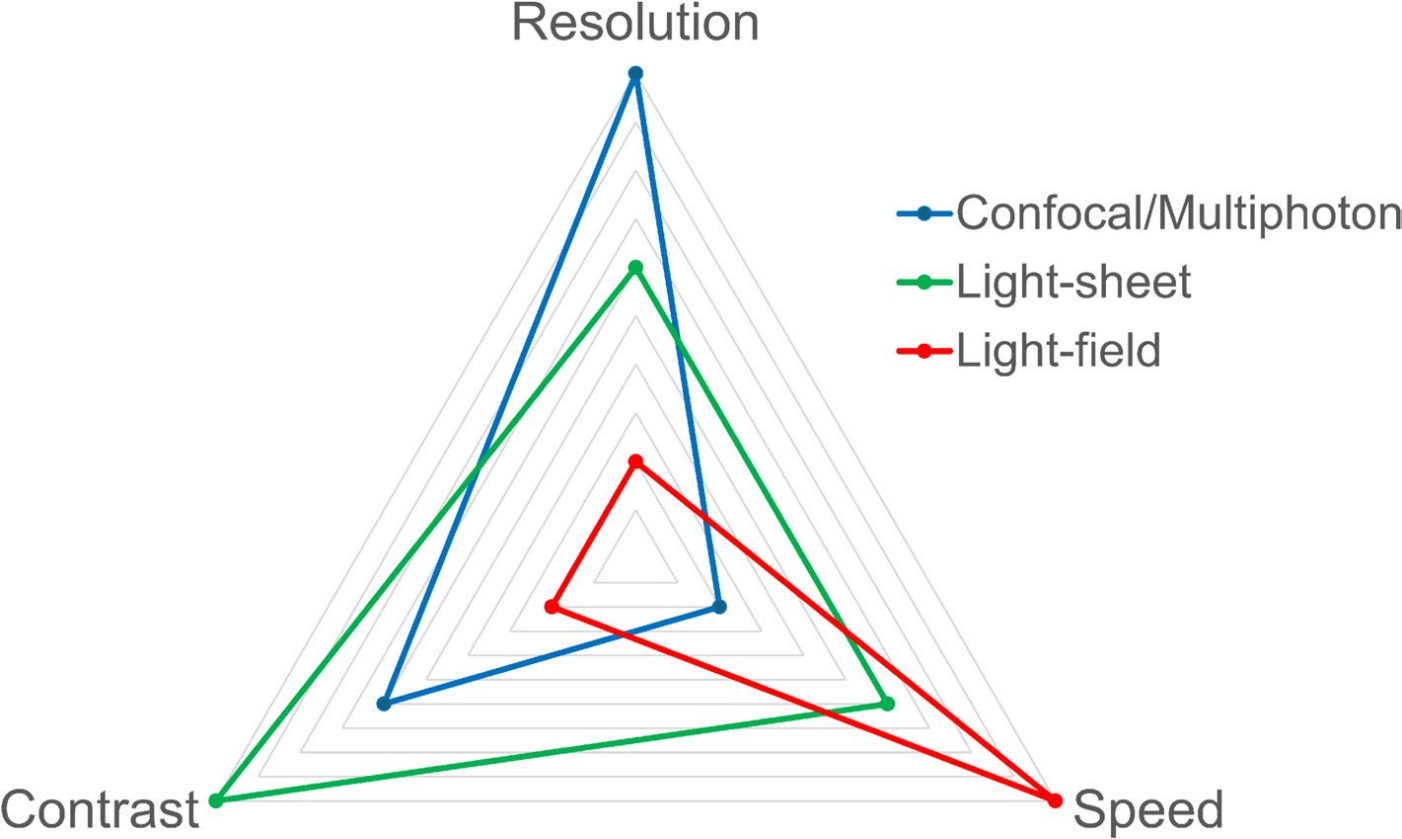
Conceptual speed–resolution–contrast framework for major 3D optical neuroimaging modalities. For this comparison, each modality is considered in its basic configuration to emphasize the physical origins of its performance trade-offs. Confocal and multiphoton microscopy are best suited for applications in which spatial resolution is the primary requirement. In these approaches, lateral resolution benefits from the combined effects of illumination and detection point-spread functions, and is therefore generally higher than in wide-field detection modalities such as light-sheet microscopy and LFM. Light-sheet microscopy balances resolution and speed through plane-wise parallel detection. Its optical sectioning is provided by confined planar illumination, which avoids the in-focus signal loss associated with confocal pinholes and suppresses out-of-focus background more effectively than nonlinear excitation in multiphoton microscopy, often leading to the highest overall image contrast. LFM achieves snapshot volumetric imaging by dividing the objective aperture across angular samples. This reduces effective spatial resolution and provides limited intrinsic optical sectioning, but enables the fastest volumetric acquisition because an entire 3D volume can be encoded in a single camera exposure. Thus, the three modalities occupy complementary operating regimes: Confocal/multiphoton microscopy for resolution-dominant imaging, light-sheet microscopy for balanced high-contrast volumetric imaging, and LFM for synchronous high-speed volumetric capture

As illustrated in Fig. 3, confocal and multiphoton approaches are preferred when spatial resolution matters the most. Light-sheet microscopy excels in image contrast and offers a balanced trade-off between resolution and speed, particularly when sample geometry permits rapid plane acquisition at tens to a few hundred volumes per second. LFM becomes most compelling when experiments demand faster synchronous volumes, when motion cannot be tamed, or when bandwidth or latency constraints make sequential scanning impractical.

### Future directions: speed-first quality, extreme temporal bandwidth, and multimodal function

Future directions for light-field neuroimaging should build on LFM’s defining advantage: synchronous snapshot volumetric capture. Advances that improve image quality are most valuable when they preserve this parallel acquisition principle rather than reintroducing sequential scanning. The greatest opportunities lie in regimes where biology demands kilohertz-and-beyond volumetric rates, low latency, and robustness to motion—conditions under which conventional confocal, multiphoton, and even state-of-the-art light-sheet methods often face difficult trade-offs among volume size, photodose, and temporal accuracy.

### Improve image quality without sacrificing snapshot speed

As discussed in Sect. 3, LFM inherently trades some image quality for speed. However, selected aspects of image quality, such as spatial resolution and optical sectioning, can be recovered to some extent while keeping acquisition fundamentally parallel. For example, supervised reconstruction methods such as virtual-scanning LFM [29] use neural networks to improve spatial resolution toward the diffraction limit by learning sample-specific priors that compensate for missing spatial-frequency information. Similarly, recent physics-based learning approaches map one-photon light-field measurements to two-photon reference volumes, improving calcium imaging in 3D brain tissue and producing contrast closer to optically sectioned two-photon microscopy [43].

These approaches highlight the power of learning-based reconstruction, but they also share an important limitation: their performance depends strongly on the training data. If the training set does not adequately represent the experimental sample, or if the model overfits to sample-specific structures, reconstruction may introduce biased or hallucinated features. Therefore, learning-based quality enhancement should be paired with uncertainty estimation and validation against independent measurements whenever possible.

In addition to learning-based quality enhancement, several hardware and physics-based strategies can improve LFM image quality while preserving its snapshot advantage. Representative approaches include SVIM [6, 7] or targeted illumination [44], which confines excitation to the volume of interest, and confocal LFM architectures, which restrict the detection volume. Notably, although SVIM and confocal LFM use scanned illumination, their detection remains camera-based, thereby preserving parallel volumetric readout while improving background suppression. Multi-view or orthogonal-view LFM [45, 46] can further recover complementary spatial-frequency information, improving axial resolution and isotropy, particularly when different views are acquired simultaneously.

Compared with purely learning-based methods, these hardware- and physics-based approaches are generally more interpretable and less dependent on training data. Although they still do not fully match the spatial resolution or contrast of confocal or light-sheet microscopy, they can narrow the performance gap while retaining LFM’s core advantage of parallel volumetric acquisition.

### Extend temporal bandwidth toward microsecond dynamics and shift from reconstruction to inference

Beyond kilohertz volumes, the next frontier is to increase temporal bandwidth while maintaining useful 3D spatial encoding. One promising direction is to combine LFM’s ability to multiplex volumetric information into a single exposure with detectors that provide much higher temporal sampling, such as streak-camera-class systems [47] or event-based sensors [34]. In such architectures, the primary output may not need to be a visually complete 3D movie at every time point. Instead, the system can be optimized for biologically relevant inference, including spike timing, event probability, source localization, propagation trajectories, synchrony, and low-latency triggers for closed-loop experiments. This reframes high-speed LFM as a task-specific volumetric sensing strategy, where success is measured by quantitative functional accuracy rather than visual appearance alone.

In this high-temporal-bandwidth regime, AI-based methods will likely be essential, but their objectives should be defined by calibrated biological inference. Reconstruction and decoding algorithms should therefore be uncertainty-aware, cross-validated, and task-specific, with performance evaluated using measurable criteria such as spike-detection accuracy, timing precision, localization error, false-positive rate, and closed-loop latency. This inference-first shift aligns with a central goal in neuroimaging: resolving the timing and causal ordering of neural events, not only their amplitudes. Microsecond-scale volumetric readout could approach intrinsic timescales relevant to axonal conduction and spike timing in single-cell physiology [48], enabling 3D mapping of propagation delays along axonal arbors, resolution of dendritic back-propagation and spikelets, measurement of high-frequency synchrony and phase relationships across microcircuits, and fast closed-loop paradigms such as spike-timing-aligned stimulation or real-time rejection of motion- and stimulation-induced artifacts in voltage recordings.

An emerging direction is AI-in-the-loop optical design**,** in which the light-field encoder and the real-time decoder are optimized jointly [36]. For example, diffractive optical elements, coded masks, or nonconventional microlens arrays could be designed to capture the most informative measurements for a specific neuroimaging task, rather than recording a generic light-field dataset for full spatial–temporal reconstruction. This approach trades exhaustive volumetric capture for an optimized measurement operator that preserves task-relevant information, such as which neurons fired, when they fired, and where they were located. Such optical-computational co-design could be especially valuable for head-mounted LFM systems in freely moving animals, where reducing optical payload, data bandwidth, and computational burden is as important as improving reconstruction quality.

### Expand LFM from intensity imaging to multimodal function: spectral, lifetime, and polarization

Snapshot 3D imaging becomes substantially more powerful when it provides more specific readouts. While conventional LFM has primarily focused on intensity-based measurements, recent work has demonstrated a clear shift toward multidimensional extensions, incorporating spectral [49–51], lifetime [52], and polarization contrast [53]. These multimodal light-field approaches introduce orthogonal biochemical and biophysical contrast without sacrificing low-latency volumetric acquisition. Spectral encoding can separate overlapping reporters (e.g., voltage, calcium, neurotransmitters) while suppressing hemodynamic or autofluorescence background; fluorescence lifetime imaging provides an intensity-independent contrast that is robust to motion and photobleaching and can report binding states or microenvironmental changes; and polarization imaging adds sensitivity to anisotropy and ordered structures such as membranes, myelinated fibers, and aligned neuronal processes.

For neuroimaging, these extensions enable reporter multiplexing, more stable quantification in behaving animals, and tighter coupling between fast neural activity and underlying physiology while preserving the speed-first motivation of light-field approaches. By adding orthogonal contrast mechanisms, they can transform LFM from a fast 3D imaging modality into a fast 3D sensing platform, providing additional functional labels that improve the decoding and interpretation of distributed neural dynamics in intact tissue.

## Conclusions

LFM is best understood as a complementary modality optimized for synchronous volumetric capture. Its performance should therefore be assessed by information throughput, latency, photon efficiency, temporal accuracy, and robustness to motion, rather than by direct comparison with quality-first modalities such as confocal, multiphoton, or light-sheet microscopy. As calcium and voltage indicators improve, and as experiments increasingly require low-latency readout and closed-loop control in behaving animals, the need for methods that can acquire volumes at biologically relevant speeds will continue to grow. With advances in optical architecture, illumination control, and AI-enabled reconstruction and inference, light-field approaches are positioned to become a practical toolkit for a growing class of high-speed neuroimaging applications.

## Data Availability

Not applicable.
